# Supplementary figures and images for: Characterization of a high-resolution breath acetone meter for ketosis monitoring
Source: PeerJ. 2020 Sep 24;8:e9969. doi: 10.7717/peerj.9969 (PMC7520093; doi:10.7717/peerj.9969)

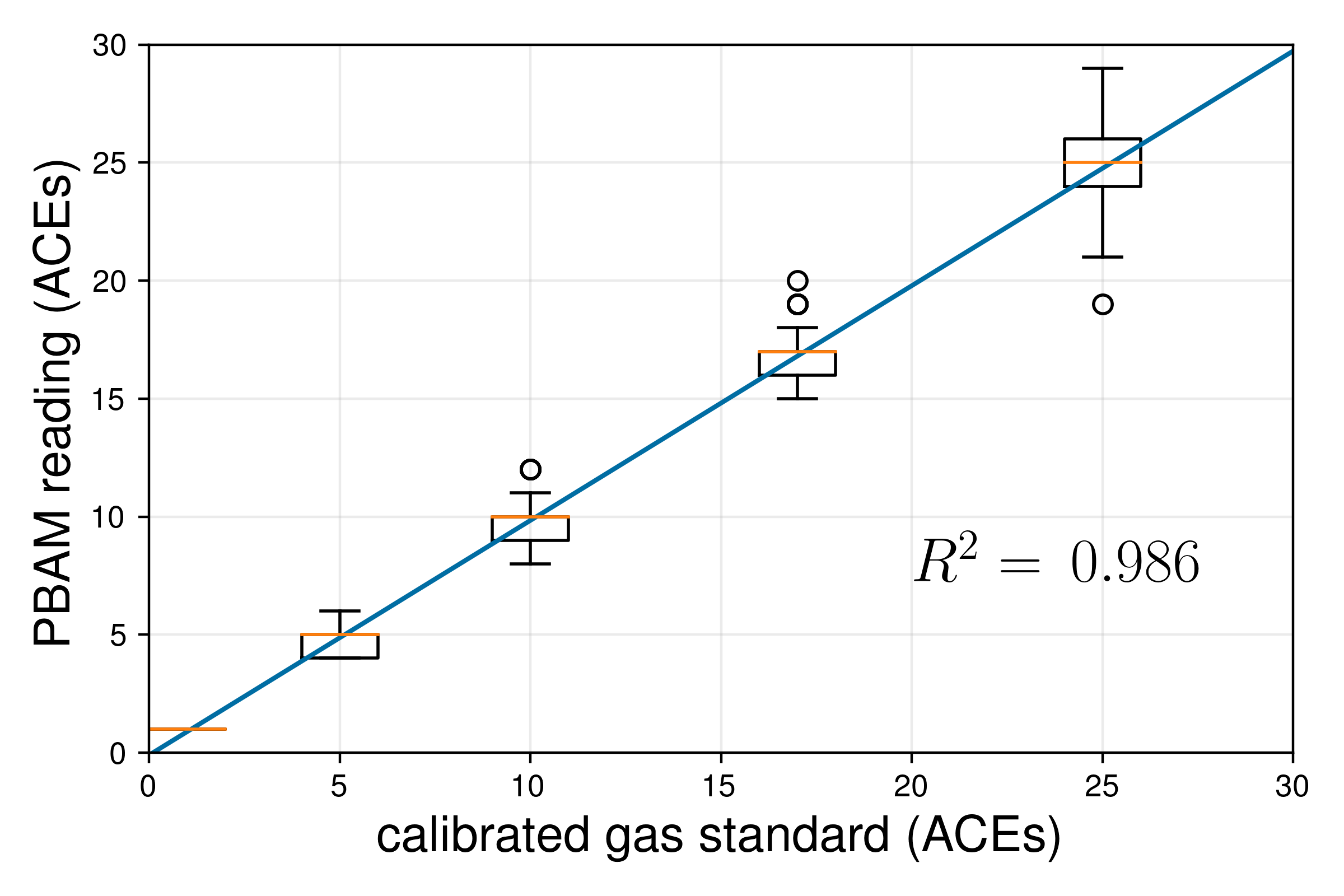

Supplement: Supplemental Information S1 — Performance of three calibrated PBAM’s against a laboratory gas standard. The readings from the PBAM and the gas standard were linearly correlated with an R2 of 0.986. The orange line indicates the median and the box edges represent the 25th quartile (Q1) and 75th quartile (Q3) for each gas concentration. The box width represents the interquartile range (IQR = Q3–Q1). The upper and lower whiskers represent the last datum less than Q3 + 1.5∗IQR and the first datum greater than Q1–1.5*IQR, respectively. Finally, the open circles represent data beyond Q3 + 1.5∗IQR and Q1 − 1.5∗IQR. [file peerj-08-9969-s001.png]

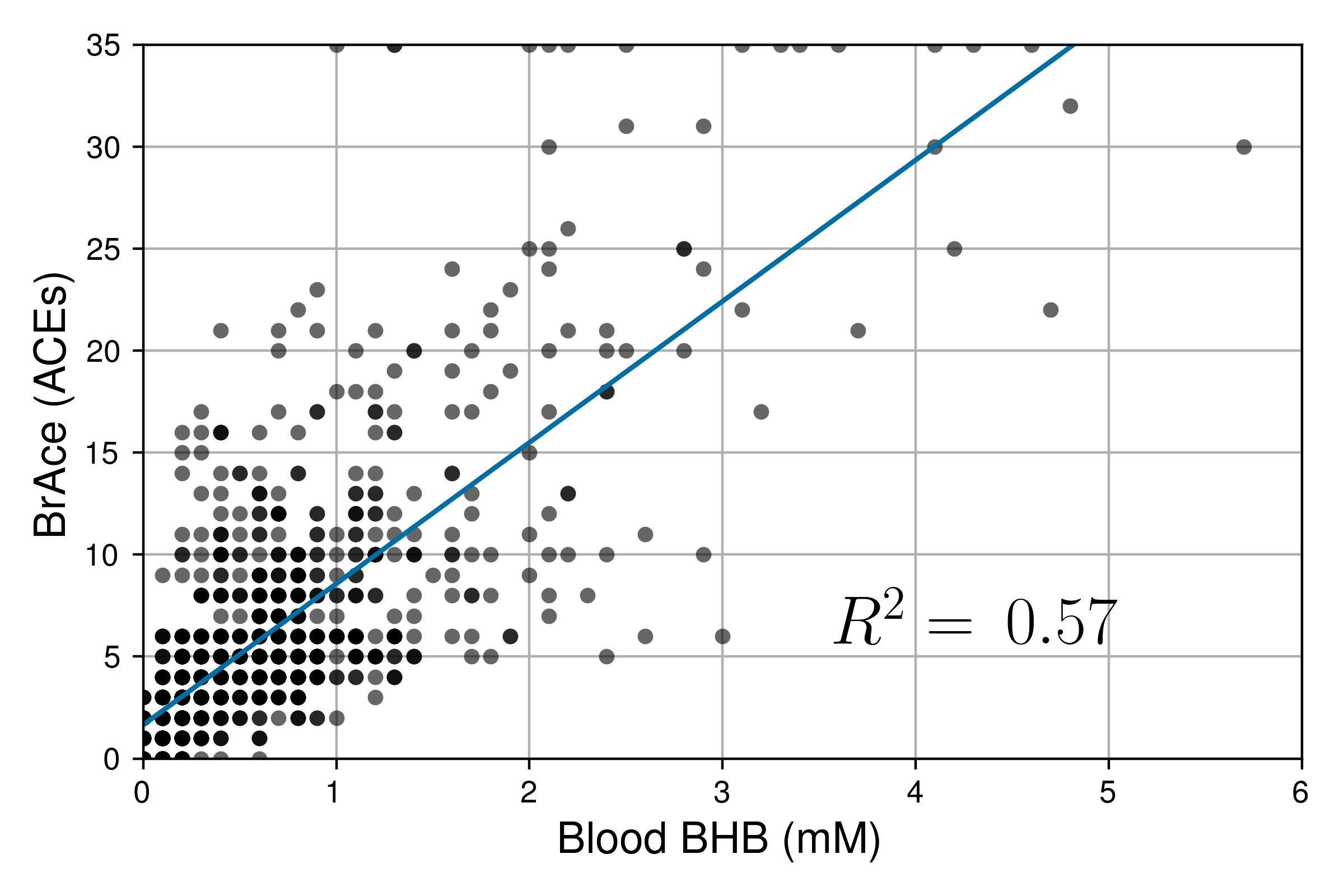

Supplement: Supplemental Information S2 — Correlation of coincident breath acetone (ACEs) and blood BHB measurements (n = 1, 214). The gray and black dots represent individual and multiple overlapping data points, respectively. BrAce and blood BHB are linearly correlated with R2 = 0.57 (P < 0.0001). This correlation coefficient is similar to literature reported values whose weighted mean is 0.64. [file peerj-08-9969-s002.png]

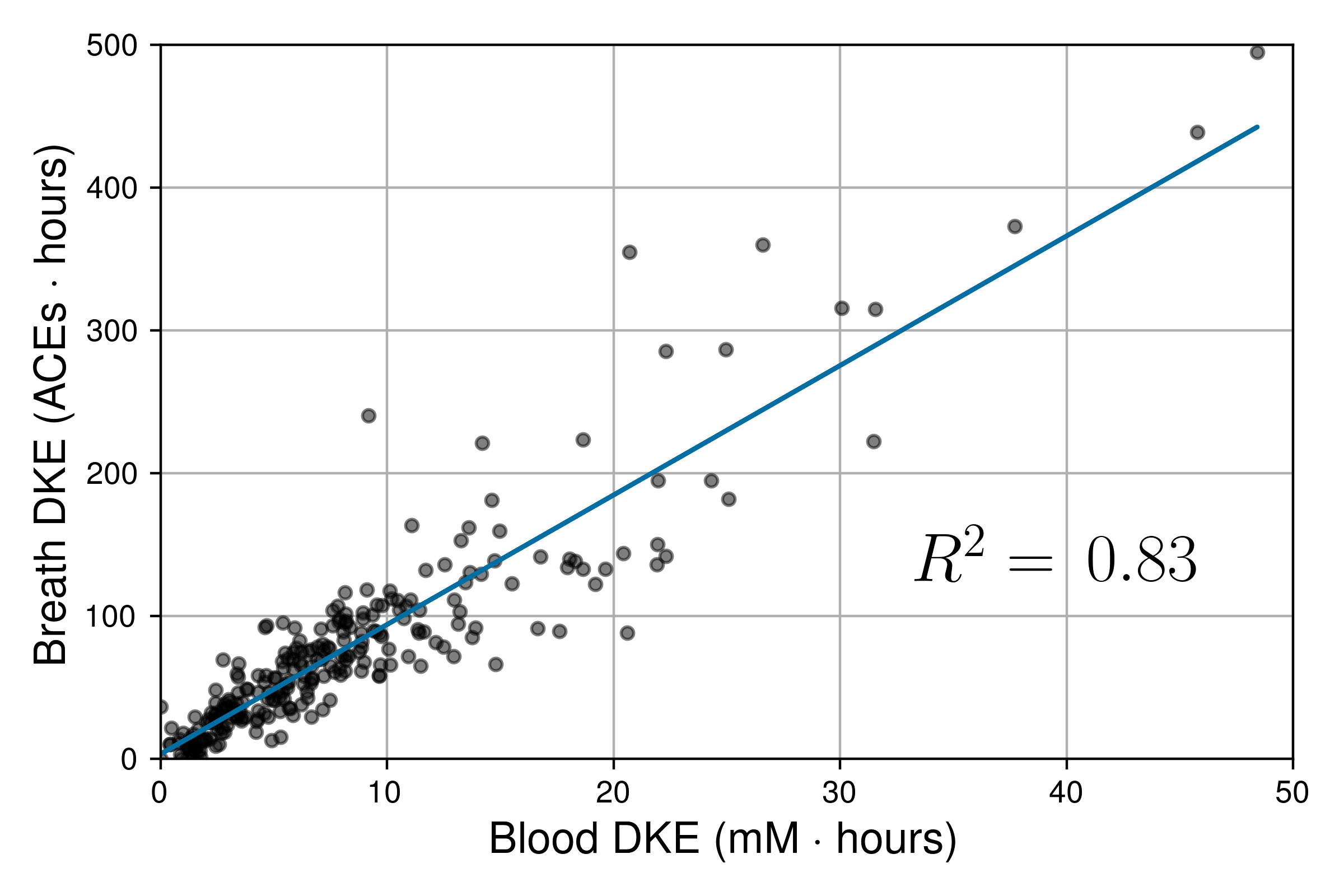

Supplement: Supplemental Information S3 — Correlation between daily ketone exposures (DKEs) as measured by breath acetone (ACEs) and blood BHB. Each data point represents one subject-day during the trial. The gray and black dots represent individual and multiple overlapping data points, respectively. Blood and breath DKEs were highly correlated (R2 = 0.83, P < 0.0001, n = 248). [file peerj-08-9969-s003.png]

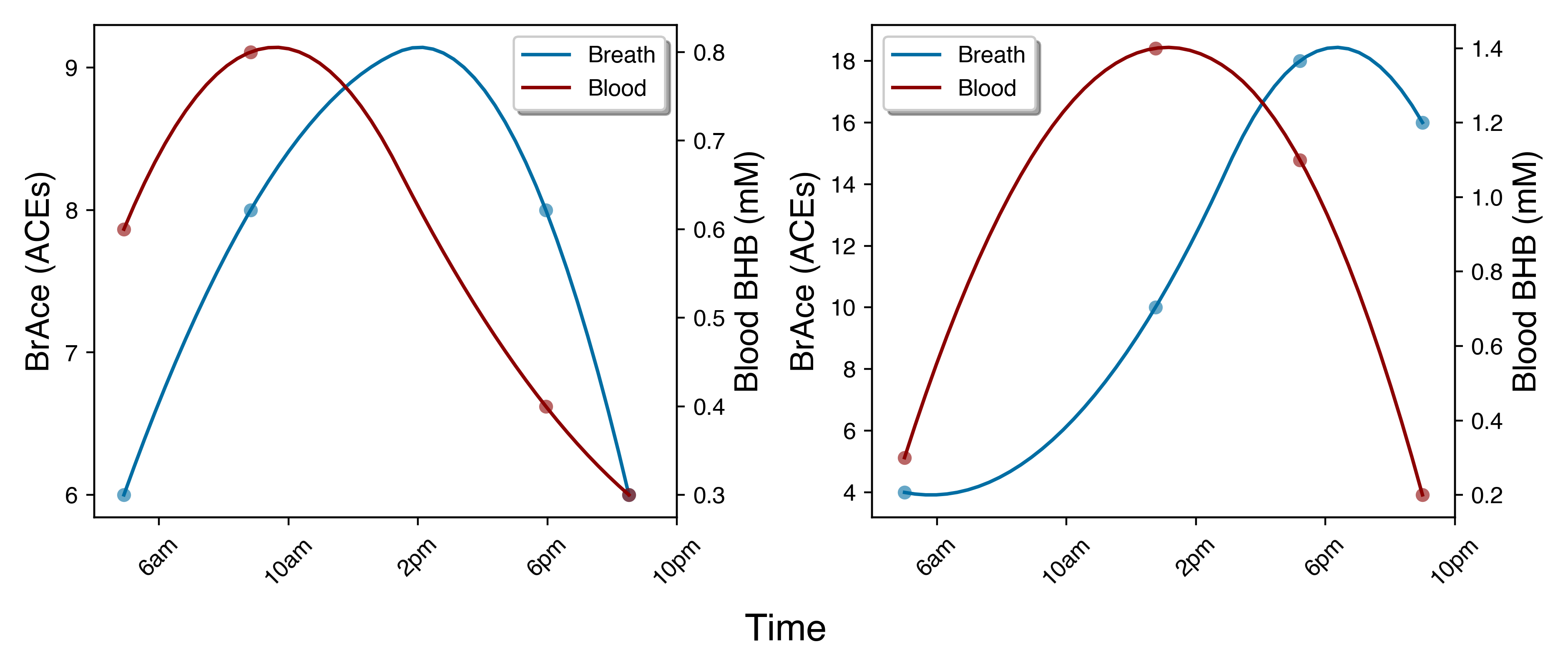

Supplement: Supplemental Information S4 — Examples of the temporal lag between blood BHB and breath acetone (ACEs). Both examples demonstrate a lag of approximately 4 hours between peak concentrations of blood BHB and breath acetone. This time lag effectively decreases the point-to-point correlation coefficient. [file peerj-08-9969-s004.png]
