# Supplementary material for: Characterization of a high-resolution breath acetone meter for ketosis monitoring
Source: PeerJ. 2020 Sep 24;8:e9969. doi: 10.7717/peerj.9969 (PMC7520093; doi:10.7717/peerj.9969)
Supplement: Supplemental Information S7 [file peerj-08-9969-s007.docx]

**Readout Health Trial Protocol
 Characterization of a Portable Solid-State Breath Acetone Testing Device for**

**Real-Time Ketosis Status and Comparison to Blood Ketone Testing**

**Protocol Number:**

**October 15, 2019**

| **Principle Investigator:** | James McCarter, MD, PhD |
| --- | --- |
|  | [jamespmccarter@gmail.com](mailto:jamespmccarter@gmail.com)  (314) 409-6891 |
|  |  |
| **Trial Coordinator:** | Trey Suntrup, PhD |
|  | [trey@readouthealth.com](mailto:trey@readouthealth.com) |
|  | (314) 603-6261 |
|  |  |
| **Sponsor:** | Readout, Inc. |
|  | 20 S. Sarah St. |
|  | St. Louis, MO 63108 |

**Purpose of the Study**

The primary purpose of this study is to characterize the performance and utility of a novel breath acetone meter developed by Readout, Inc., a medical device startup company based in St. Louis, Missouri. Ketone bodies (“ketones”) acetoacetate, beta-hydroxybutyrate and acetone are products of fatty acid metabolism that circulate in the blood. Acetone is volatile and can be detected non-invasively in breath reflecting ketosis status.

Objective 1: to determine the relationship between breath acetone (BrAce) and blood beta-hydroxybutyrate (BHB) in multiple subjects and time points.

Objective 1a: to assess the correlation between BrAce and blood BHB concentrations by linear regression

Objective 1b: to assess the ability of the BrAce measurement to predict group classification determined by standard blood BHB thresholds.

Objective 1c: to determine the BrAce thresholds that most accurately predict group classification determined by standard blood BHB thresholds.

Objective 1d: to compute sensitivity and specificity of the BrAce test using thresholds determined in Objective 1c with blood BHB as the reference.

Objective 2: to characterize the reliability of the Readout BrAce device in determining acetone concentration by performing test-retest measurements and analyzing repeatability.

Exploratory objective 1: to assess the utility of a single ketone measurement compared to multiple measurements throughout the day.

Exploratory objective 2: to explore the time dynamics of blood BHB and BrAce to determine whether there is a significant temporal relationship between changes in these two ketones.

Exploratory objective 3: to compare the full-day ketone exposure measured by BrAce with the full-day ketone exposure measured by blood BHB.

Exploratory objective 4: to obtain qualitative feedback on device function and user experience with the BrAce and blood ketone meters.

**Background**

Interest in nutritional ketosis as a therapy has grown rapidly in the last several decades. Between 2000 and 2018 the annual number of academic publications studying the ketogenic diet has increased from 38 to 330. Utilized as a therapy for epilepsy since the 1920s, the ketogenic diet is currently under investigation as a potential treatment for a wide variety of diseases including Alzheimer’s, Parkinson’s, type-1 diabetes, fatty liver disease and cancer. Perhaps the most promising recent development is the demonstration that a ketogenic diet can effectively reverse type-2 diabetes (T2D). In a one-year controlled study,^1^ an intensive ketogenic dietary intervention resulted in a 60% diabetes reversal rate, defined as achieving glycemic control without the need for diabetes-specific medication. Subjects following a ketogenic diet reduced their hemoglobin A1C by an average of 1.3%, lost an average of 30 pounds and improved their calculated 10-year cardiovascular risk by 12%.^2^ This study and over 30 other randomized controlled trials led the American Diabetes Association to recognize the benefits of low-carbohydrate and very-low-carbohydrate diets in their 2019 Standards of Care publication for the first time.^3^

The ketogenic diet consists of very high fat (70-80% of daily calories), moderate protein (15-20%) and very low carbohydrate (5-10%). This macronutrient composition causes the body to switch from a glucose burning state to a fat burning state called ketosis (defined as a blood BHB concentration greater than 0.5 millimolar). In addition to a low-carbohydrate, high-fat diet, ketosis can also be achieved with caloric restriction or prolonged fasting to metabolize body fat in response to a net energy deficit. Research has uncovered a variety of therapeutic benefits associated with the state of ketosis including weight loss,^4,5^ restoration of insulin sensitivity,^6^ and a potent anti-inflammatory response.^7^ Given that these beneficial effects are present when an individual achieves and sustains a certain level of ketosis, it is important to be able to quickly and easily check ketone levels throughout the day.

An individual can check the presence and degree of ketosis by measuring one of three ketone bodies. As fatty acids enter the liver, they are converted via acetyl CoA to BHB and acetoacetate in the hepatocyte mitochondria. Circulating acetoacetate is converted to acetone by non-enzymatic decarboxylation. Acetoacetate is excreted in the urine and is measured using urinary dip sticks; circulating BHB is measured in the blood using a finger prick and test strip system (similar to blood glucose); acetone is excreted in the breath and is measured using various gas sensing devices. Urinary acetoacetate measurements are non-invasive but provide only a rough indication of ketosis. Blood BHB tests are widely considered to be the “gold standard” for ketone measurement due to their accuracy. However, finger-stick blood tests are uncomfortable and awkward to perform and require expensive consumable test-strips for each test, which limits their frequency of use.

Breath acetone (BrAce) has been measured for decades in the lab using mass spectrometers, gas chromatographs and other systems. These devices have been used to demonstrate the high degree of correlation between blood BHB and breath acetone in a clinical setting.^8,9^ Only recently have compact, point-of-care BrAce devices emerged as feasible alternatives to laboratory methods. These devices have the strong advantage of being simultaneously portable and non-invasive. However, portable BrAce devices have yet to achieve market traction among ketone testers due to their poor accuracy and repeatability.

Readout, Inc. was founded in 2018 to create portable, accurate and non-invasive breath technologies to support chronic disease management and consumer health. The company’s first product is a solid-state breath acetone device that addresses the accuracy and repeatability problems that have hindered existing BrAce devices. A large component of the run-to-run variability in legacy BrAce devices is due to the dependence of BrAce concentration on the portion of the exhaled breath that is sampled. In particular, the concentration of BrAce is highest when sampling alveolar air at the end of exhalation. Alveolar gas is in equilibrium with capillary blood and therefore best reflects acetone in pulmonary circulation. Without standardizing the portion of the exhaled breath that is measured, readings often suffer from large run-to-run variability.

The Readout device overcomes these limitations by using highly selective sensors and a technique known as end-of-breath sampling. A combination pressure sensor and pumping mechanism ensures that only the final portion of the user’s breath derived from the alveoli is sampled during a measurement. This simultaneously improves both repeatability and accuracy. Additionally, because the Readout device has no consumables (e.g. test strips), there is no cost associated with each additional test. Having a portable, non-invasive and accurate ketone measurement tool will allow patients and consumers following a ketogenic diet to obtain quick and frequent feedback on how their dietary and lifestyle choices affect their ketone levels. The company believes that such feedback will improve dietary adherence and ultimately disease outcomes.

The Readout device is scheduled for commercial release in late 2019 as a Class I exempt device. There are no disease management claims in the intended use statement; rather the device is intended to be used only to measure ketones. FDA registration will occur in October 2019.

**Criteria for Subject Selection**

This pilot study will consist of 22 subjects in two cohorts: 18 subjects already following a ketogenic diet or low-carbohydrate diet will be recruited along with 4 who follow a standard high-carbohydrate (mixed macronutrient) diet. The purpose of the high-carbohydrate cohort is to include data capture for zero or very low ketosis levels. We will recruit both male and female subjects over the age of 18 into both cohorts of the study. All ethnic groups will be considered, and no vulnerable subjects will be selected. Written informed consent will be obtained from each subject before study participation. No subjects will be asked to make dietary changes.

Inclusion criteria:

- Ketogenic diet cohort: currently following a ketogenic or low-carbohydrate diet defined as less than 30 grams per day (ketogenic) or less than 100 grams per day (low-carbohydrate) as estimated by the individual. Subjects must have been following the diet before the beginning of the study period and must continue with the diet throughout the duration of the trial.
- High-carbohydrate cohort: currently following a diet that does not restrict dietary carbohydrate. Carbohydrate consumption should be greater than 100 grams per day as estimated by the individual. Subjects must have been following the diet before the study period and must continue with the diet throughout the duration of the trial.
- Daily access to an iPhone or Android mobile device.

Exclusion criteria:

- Type-1 diabetes
- Insulin-dependent type-2 diabetes
- History of diabetic ketoacidosis
- Currently taking Warfarin or other blood thinners
- Currently taking a sodium-glucose cotransporter-2 (SGLT2) inhibitor
- Currently taking Disulfiram
- Unwilling to maintain their diet during the study period
- Unwilling to test blood and breath ketones five times per day
- Non-English speaking
- Persons who are unable or unwilling to follow the study protocol or who, for any reason, the research team considers not an appropriate candidate for this study, including non-compliance with screening appointments or study visits.

**Methods and Procedures**

All subjects will be provided with a Readout breath acetone device and an Abbott Precision Xtra blood ketone meter with test strips and supplies (the Precision Xtra is an FDA-approved commercially available device and the lancets and alcohol swabs are commercially available medical supplies). Subjects will also be provided with an iOS mobile app that will sync with the Readout device via Bluetooth and automatically read and store breath ketone measurements. Subjects will manually submit their blood ketone values using the same mobile application.

The trial coordinator will provide verbal and written study instructions and in-person individual training on the use of both devices during an initial visit. Subjects will be instructed to measure their ketones at least five times per day every day for two weeks at the following times:

1. First thing in the morning (before eating or using toothpaste/mouthwash)
2. Right before lunch (e.g. 11am – 12pm)
3. Approximately 2 hours after eating lunch (e.g. 2 – 4pm)
4. In the evening before dinner (e.g. 5 – 7pm)
5. In the evening after dinner (e.g. 8 – 9pm)

Times will vary by person and day based on each individual’s daily mealtimes. Five measurements per day are expected no matter the number of meals per day, e.g. during fasting or time restricted feeding. (Additional testing for participants interested in further exploring their ketone levels will be allowed including additional measures of BrAce without blood BHB testing.)

At each of the scheduled times, subjects will take two back-to-back breath ketone measurements and one blood ketone measurement. Additional breath ketone measurements at other times during the day will be permitted at the subject’s discretion. The total estimated daily time required for all measurements is 35 minutes (7 minutes per testing session).

Because subjects are selected according to the diet they are following before the study period, they will be given very little dietary guidance. Subjects in the ketogenic diet arm will be encouraged to maintain ketone levels at or above 0.5 millimolar (mM) blood BHB equivalent. Subjects in the high-carbohydrate cohort will be instructed not to adjust their diet based on their ketone readings. This is to prevent behavior change due to non-ketogenic subjects becoming motivated to increase their ketone levels.

For the majority of subjects, the ketone measurements performed in this study will be exclusively for research purposes. However, some subjects may regularly measure ketone levels as part of their normal routine outside of the study.

**Data Storage and Confidentiality**

The study will be conducted in compliance with HIPAA (The Health Insurance Portability and Accountability Act). In order to protect patient confidentiality, each study subject will be randomly assigned a unique numerical code that will serve as their direct identifier. Each subject’s ketone data, time stamp and numerical identifier will be collected via mobile app and uploaded to Readout’s Amazon Web Services (AWS) database. Data contained in the AWS database is accessible only with Readout authentication credentials, and the data will be encrypted at rest. The code key linking numerical identifiers with other direct identifiers such as name and address will be stored on the drives of the trial PI and coordinator and kept under password protection. Only the principle investigator and the trial coordinator will have access to the code key.

**Data Analysis Methods**

Objective 1:

Objective 1a: we will assess the correlation between BrAce (parts per million, ppm) and blood BHB (mM) using linear ordinary least squares regression analysis and the coefficient of determination (R^2^). Correlations will be computed between individual BHB (independent variable) and BrAce (dependent variable) readings taken at the same time point. We will use a one-sided t-statistic to test the null hypothesis that the slope of the regression line (b in y = a + b*x) is zero. We will determine the p-value in a t-test lookup table. We will also determine the F-statistic to test the likelihood that the observed relationship between the dependent and independent variables occurs by chance. The p-value cutoff for significance will be 0.05. If inspection of data suggests that a non-linear approach is required, a non-linear regression analysis will be performed. We will repeat this analysis using units of ACEs (a proprietary unit of measure developed by Readout) for BrAce and units of mM for blood BHB.

Objective 1b: we will assess the ability of the BrAce measurement to predict classification in groups determined by standard blood BHB concentration thresholds by calculating sensitivity and specificity of the BrAce test at predetermined thresholds. Example cutoff points are blood BHB values of 0.3 mM (slightly elevated ketones), 0.5 mM (nutritional ketosis), 1.0 and 1.5 mM (higher levels of nutritional ketosis).

Objective 1c: we will determine the BrAce thresholds that most accurately predict classification determined by standard blood BHB thresholds by performing a Receiver Operating Curve (ROC) analysis at the group level. Example thresholds are blood BHB values of 0.3 mM (slightly elevated ketones), 0.5 mM (nutritional ketosis), 1.0 and 1.5 mM (higher levels of nutritional ketosis).

Objective 1d: we will compute the sensitivity and specificity of the BrAce test using the thresholds determined by Objective 1c with blood BHB (mM) as the reference.

Objective 2: we will to characterize the test-retest reliability of the Readout BrAce device using two methods: first, we will calculate alpha reliability coefficients for each subject using the repeated measurements at each measurement point. Second, we will compute coefficients of variation for each measurement point using repeated BrAce readings.

Exploratory objective 1: we will assess the utility of a single BrAce measurement compared to multiple measurements throughout the day by comparing a single measurement for a given user with the time-weighted average of all measurements taken that same day (12 AM on day 1 to 12 AM on day 2). The difference between the single measurement and time-weight average will be plotted as a probability distribution at both the individual and the group levels. Finally, the probability that a single measurement differs from the time-weighted average of all measurements during the same day will be computed for various difference thresholds.

Exploratory objective 2: we will explore the time dynamics of blood BHB versus BrAce by performing a correlation calculation for various time shifts between the two measurement methods. In preliminary testing, we have observed a slight time delay in peak and trough BrAce levels following peak and trough BHB of approximately 1 to 2 hours. Identifying the time shift that leads to the correlation coefficient improvement will provide information about the probable time lag between responses in blood BHB and BrAce. We will perform this analysis at both the individual and the group level. This analysis will leverage additional testing performed by participants beyond the obligatory 5 times per day including breath tests that are done without corresponding blood tests.

Exploratory objective 3: we will compare the full-day ketone exposure as measured by BrAce with the full-day ketone exposure as measured by blood BHB (12 AM on day 1 to 12 AM on day 2) by first performing linear interpolation between data points on a given day and then calculating the daily area under the curve (AUC) for BrAce and blood BHB. We will use two methods to compare calculated BrAce and blood BHB AUCs: first, we will determine whether there is a correlation between blood and breath AUC by performing linear regression analysis. Second, we will calculate the difference between daily blood and breath AUC and plot the difference as a probability distribution at both the individual and the group level. The probability that blood and breath AUC differ will then be computed for various difference thresholds.

Exploratory objective 4: we will gather qualitative feedback on device function and user experience via exit surveys. Surveys will include questions related to ease of use and the utility of frequent biomarker feedback. For trial participants attempting to lose weight during the two week testing period, we will inquire into their perception of ketone tracking as aiding this objective.

**Risk/Benefit Assessment**

We anticipate that the potential risks associated with participation in this study are minor and warrant a minimal risk classification. The primary risk associated with this study is loss of confidential biomarker data that is gathered during the study. This risk is being mitigated by procedures described in the section on Data Storage and Confidentiality. In addition, all personnel involved in the design and conduct of this research project will receive the required education on the protection of human research participants prior to the start of this project.

Research participants may benefit from this study by gaining unique and individualized insight into the effects of dietary and lifestyle choices on their metabolism.

**Subject Identification, Recruitment and Consent**

Research subjects will be identified from within the personal and professional networks of Readout employees and advisors. Subjects that are geographically close to the trial investigators will be prioritized in order to facilitate training and technical support.

The principle investigator and trial coordinator will be authorized to obtain informed consent from research subjects. The objectives of the study, all experimental procedures, all of the requirements for participation, and any possible discomforts and risks and benefits of participation will be clearly explained in writing and orally, in lay terms, to the participant by the PI and/or trial coordinator. After all questions have been answered, and the participants have fully informed orally and in writing that they are free to withdraw from the study at any time with no bias or prejudice, and agree to participate, written informed consent, approved by Western Institutional Review Board (WIRB), will be obtained. Only subjects that have the capacity to provide informed consent will be considered.

All trial subjects who complete the entire two week study and remain compliant with daily measurements will receive a $100 cash stipend and a Readout breath acetone device for ongoing personal use.

**References**

1. Hallberg, S. J. *et al.* Effectiveness and Safety of a Novel Care Model for the Management of Type 2 Diabetes at 1 Year: An Open-Label, Non-Randomized, Controlled Study. *Diabetes Therapy* **9**, 583–612 (2018).

2. Bhanpuri, N. H. *et al.* Cardiovascular disease risk factor responses to a type 2 diabetes care model including nutritional ketosis induced by sustained carbohydrate restriction at 1 year: an open label, non-randomized, controlled study. *Cardiovasc Diabetol* **17**, 56–56 (2018).

3. Evert, A. B. *et al.* Nutrition Therapy for Adults With Diabetes or Prediabetes: A Consensus Report. *Diabetes Care* dci190014 (2019). doi:10.2337/dci19-0014

4. Reduction in the Incidence of Type 2 Diabetes with Lifestyle Intervention or Metformin. *N Engl J Med* **346**, 393–403 (2002).

5. Van Gaal, L. & Scheen, A. Weight Management in Type 2 Diabetes: Current and Emerging Approaches to Treatment. *Diabetes Care* **38**, 1161 (2015).

6. Boden, G., Sargrad, K., Homko, C., Mozzoli, M. & Stein, T. P. Effect of a Low-Carbohydrate Diet on Appetite, Blood Glucose Levels, and Insulin Resistance in Obese Patients with Type 2 Diabetes. *Annals of Internal Medicine* **142**, 403–411 (2005).

7. Shimazu, T. *et al.* Suppression of Oxidative Stress by β-Hydroxybutyrate, an Endogenous Histone Deacetylase Inhibitor. *Science* **339**, 211 (2013).

8. Anderson, J. C. Measuring breath acetone for monitoring fat loss: Review. *Obesity (Silver Spring)* **23**, 2327–2334 (2015).

9. Musa-Veloso, K., Likhodii, S. S. & Cunnane, S. C. Breath acetone is a reliable indicator of ketosis in adults consuming ketogenic meals. *The American Journal of Clinical Nutrition* **76**, 65–70 (2002).
