# Supplementary material for: Characterization of a high-resolution breath acetone meter for ketosis monitoring
Source: PeerJ. 2020 Sep 24;8:e9969. doi: 10.7717/peerj.9969 (PMC7520093; doi:10.7717/peerj.9969)
Supplement: Supplemental Information S8 [file peerj-08-9969-s008.pdf]

In addition to parts per million (ppm), the PBAM also reports breath acetone results in units called ACEs, which are designed to translate parts per million (ppm) of breath acetone into a blood BHB equivalent. As blood BHB and breath acetone are enzymatically and non-enzymatically converted from AcAc, and breath acetone is additionally dependent on blood-gas partitioning, the relationship between breath acetone and BHB is nonlinear. Based on the literature and our own data the relationship can be described by a function of the form:

$$1\,\text{ACEs} = 10 * \text{BHB} = Ax^B + C,$$

where x is breath acetone in ppm and A, B and C are device-independent coefficients.

Figure 1 shows the performance of three calibrated PBAMs upon repeated exposure to the laboratory acetone standards in units of ACEs.

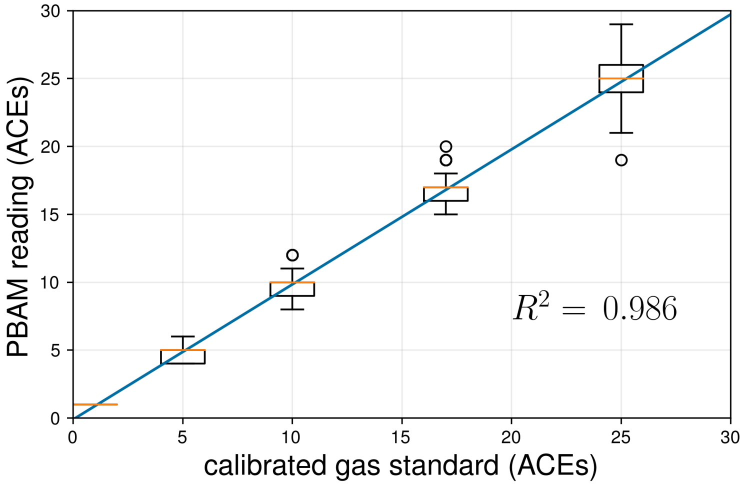

Figure 1. Performance of three calibrated PBAM's against a laboratory gas standard. The readings from the PBAM and the gas standard were linearly correlated with an R^2 of 0.986. The orange line indicates the median and the box edges represent the 25th quartile (Q_1) and 75th quartile (Q_3) for each gas concentration. The box width represents the interquartile range (IQR = $Q_3 - Q_1$).The upper and lower whiskers represent the last datum less than $Q_3 + 1.5 * \text{IQR}$ and the first datum greater than $Q_1 - 1.5 * \text{IQR}$, respectively. Finally, the open circles represent data beyond $Q_3 + 1.5 * \text{IQR}$ and $Q_1 - 1.5 * \text{IQR}$.

Figure 2 shows the correlation between coincident breath acetone (ACEs) and blood BHB measurements (n=1,214).

Figure 3 shows the correlation between DKEs for breath acetone (ACEs) and blood BHB (n=248).

Figure 4 demonstrates the temporal lag between blood BHB and breath acetone (ACEs).

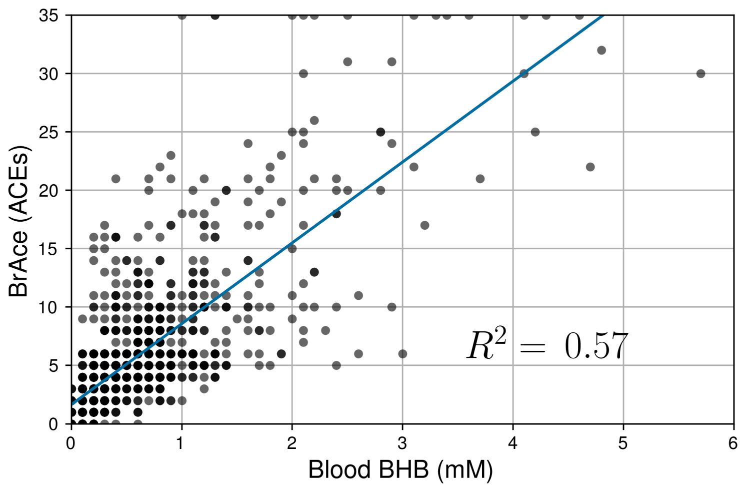

Figure 2. Correlation of coincident breath acetone (ACEs) and blood BHB measurements (n=1,214). The gray and black dots represent individual and multiple overlapping data points, respectively. BrAce and blood BHB are linearly correlated with $R^2 = 0.57$ (P<0.0001). This correlation coefficient is similar to literature reported values whose weighted mean is 0.64.

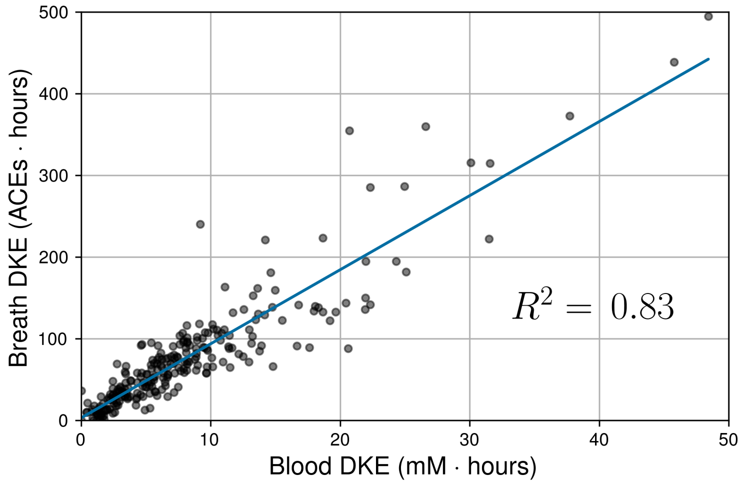

Figure 3. Correlation between daily ketone exposures (DKEs) as measured by breath acetone (ACEs) and blood BHB. Each data point represents one subject-day during the trial. The gray and black dots represent individual and multiple overlapping data points, respectively. Blood and breath DKEs were highly correlated ($R^2 = 0.83$, P<0.0001, n=248).

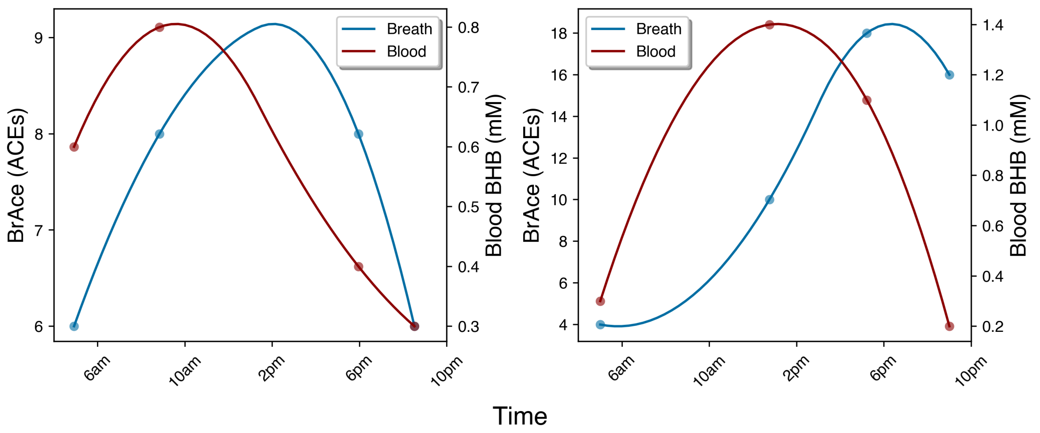

Figure 4. Examples of the temporal lag between blood BHB and breath acetone (ACEs). Both examples demonstrate a lag of approximately 4 hours between peak concentrations of blood BHB and breath acetone. This time lag effectively decreases the point-to-point correlation coefficient.
